# Clinical management and associated costs for moderate and severe Alzheimer’s disease in urban China: a Delphi panel study

**DOI:** 10.1186/s40035-015-0038-9

**Published:** 2015-08-20

**Authors:** Xin Yu, Shengdi Chen, Xiaochun Chen, Jianjun Jia, Chunhou Li, Cong Liu, Mondher Toumi, Dominique Milea

**Affiliations:** Institute of Mental Health, Peking University Sixth Hospital, Huayuanbeilu 51, Haidian District, Beijing, 100191 China; Department of Neurology, Ruijin Hospital affiliated to Shanghai Jiao Tong University School of Medicine, Shanghai, China; Fujian Institute of Geriatrics, Union Hospital of Fujian Medical University, Fuzhou, China; Department of Geriatric Neurology, Chinese PLA General Hospital, Beijing, China; Medical Services Department, Peking Union Medical College Hospital, Beijing, China; Beijing Tongren Hospital, Capital Medical University, Beijing, China; University of Aix-Marseille, Marseille, France; Lundbeck Singapore, Singapore, Singapore

## Abstract

**Background:**

Healthcare resource utilisation for Alzheimer’s disease (AD) in China is not well understood. This Delphi panel study aimed to describe the clinical management pathways for moderate and severe AD patients in urban China and to define the amount and cost of healthcare resources used.

**Methods:**

A panel of 11 experts was recruited from urban China to participate in two rounds of preparatory interviews. In the first round, 9 physicians specialised in dementia gave a qualitative description of the clinical management of AD patients. In the second round, 2 hospital administrators were asked about the cost of AD management and care. Results from the interviews were discussed by the experts in a Delphi panel meeting, where consensus was reached on quantitative aspects of AD management, including the rate of healthcare resource utilisation, the respective unit costs and caregiving time.

**Results:**

Interviewees reported that mild AD is under-recognised in China; most patients are diagnosed with moderate to severe AD. Loss of independence and agitation/aggression are the main drivers for healthcare resource utilisation and contribute to a heavier caregiver burden. It was estimated that 70 % moderate AD patients are independent/non-aggressive at the time of diagnosis, 15 % are independent/aggressive, 10 % are dependent/non-aggressive, and 5 % are dependent/aggressive. Dependent/aggressive AD patients are more likely to be hospitalised (70–90 %) than accepted in a nursing home (0–20 %), while the opposite is true for dependent/non-aggressive patients (5–35 % for hospitalisation vs. 80 % for nursing home). Independent AD patients require 1–3 hours/day of caregiver time, while dependent patients can require up to 12–15 hours/day. Experts agreed that AD complicates the management of age-related comorbidities, found in 70–80 % of all AD patients, increasing the frequency and cost of hospitalisation.

**Conclusions:**

The Delphi panel approach was an efficient method of gathering data about the amount of healthcare resources used and associated costs for moderate and severe AD patients in urban China. The results of this study provide a useful source of information for decision makers to improve future healthcare policies and resource planning, as well as to perform economic evaluations of AD therapies.

## Background

Alzheimer’s disease (AD) is a neurodegenerative disorder characterised by gradual memory impairment, agnosia and decline in cognitive function. In addition, AD patients frequently present with behavioural changes and neuropsychiatric symptoms (NPS), such as apathy, depression, agitation, aggression, insomnia, impaired motor coordination and delusions [1, 2]. Progressive deterioration impacts on personal autonomy, with patients becoming increasingly reliant on caregivers [3, 4]. In the final stages of AD, patients lose their ability to communicate, fail to recognise loved ones, become bedridden and require continuous care [4]. AD is most prevalent among people aged 70–85 years and affects around twice as many women as men [3, 5]. Approximately 5.7 million people in China are estimated to have AD, more than any other country in the world [5].

AD diagnosis is usually made by primary care physicians upon examining the patient’s medical history in consultation with a close relative or caregiver, followed by cognitive tests and physical examinations. Brain imaging procedures can also be performed to identify brain changes responsible for the symptoms [4]. Although there is no effective cure for AD, pharmacologic agents, such as acetylcholinesterase inhibitors and N-methyl-D-aspartate (NMDA) receptor antagonists, can assist in symptom management by slowing the progressive decline in cognitive function [4, 6, 7]. However, reports suggest that only 10 % of individuals with dementia in China are diagnosed, of which 21 % are prescribed medication [8–10].

The increasing life expectancy in China has led to a growing concern over age-related diseases, of which AD is predicted to have the greatest clinical, societal and economic impact [5, 11]. In 2009, the total yearly cost of dementia in China was estimated to be approximately 6.4 billion USD [11]. Thus, adequate provision of healthcare resources to AD is a growing problem.

The cost of AD care depends on disease severity and therefore varies greatly [12, 13]. Since the majority of AD patients in China (96 %) are cared for at home by family members [14], the disease costs are associated with a substantial social burden—caregivers frequently report a high level of emotional and physical stress [15–17]. Although the economic impact of AD has been well-documented in numerous countries [18–24], few cost-of-illness studies for AD have been performed in China. To our knowledge, only one study has investigated the healthcare resources used by AD patients in China [25]. This survey, conducted in 2005–2006, considered 67 AD patients (13 mild, 37 moderate and 16 severe) in one Shanghai hospital. Annual costs, estimated by retrospective interviews, were significantly associated with patients’ degree of cognitive impairment and autonomy. Direct costs were similar between the different disease stages, while indirect costs (mainly informal caregiving) increased drastically with AD severity.

Healthcare policies and patient management in China vary widely between different provinces and among hospitals of the same province. Despite recent progress, there are still substantial differences in the type and cost of healthcare services provided, medical insurance schemes and government investment in healthcare infrastructure [26]. As a consequence, the pattern of healthcare resource utilisation for AD in China is not well understood, preventing an adequate comparison of the cost-effectiveness of pharmacological interventions.

When published information on a particular question is limited, the Delphi panel method can be used to obtain expert-validated evidence. In this type of study, a panel of experts is interviewed about the topic under research, and the combined responses are anonymised and shared with all participants. The panel of experts then review their initial responses in light of group-wide choices until consensus is reached [27].

Due to the paucity of data on the healthcare resources needed for AD in China, we conducted a Delphi panel study to describe the typical clinical management pathways for AD patients diagnosed by physicians specialised in dementia care, focussing on urban areas of China where pharmacological treatments are available. In addition, we evaluate the impact of disease severity and symptoms on healthcare resource utilisation and amount of caregiving required. Thirdly, we determine the unit costs of the healthcare resources associated with the management of AD. The information obtained was used to develop a health economic model to evaluate the cost-effectiveness of pharmacological treatment for moderate and severe AD patients in China [28].

## Methods

### Study overview

The process for this Delphi panel study started with two preparatory rounds of interviews, to perform a qualitative evaluation of the clinical management of moderate and severe AD patients in China. In the first round of interviews, experts were asked to describe the clinical aspects of AD diagnosis and management. In the second round, experts were asked to estimate the economic costs associated with moderate and severe AD. All information gathered was anonymised and combined for discussion in the Delphi panel meeting, including diverging opinions and unclear answers.

The interviews were followed by a final consensus meeting, where the experts discussed their answers to reach consensus on quantitative aspects of AD management, including the distribution of AD patients across a range of health states at diagnosis, the impact of disease severity and symptoms on the amount of healthcare resources required, and the unit cost of each healthcare resource. The values agreed upon by the experts were to be used as inputs in a health economic model, designed to evaluate the clinical and economic outcomes of pharmacological treatment of moderate and severe AD patients in China [28].

### Participants

Nine physicians specialising in AD were selected to be interviewed on the clinical management of disease. Physicians were recruited based on their level of experience (more than 10 years of clinical practice), their expertise in treating AD patients and their understanding of the impact of healthcare policies on patient management. The physicians selected worked in a range of healthcare institutions, including general and psychiatric wards, routinely met patients covered by different reimbursement schemes and were recruited from three tier 1 cities in China: Beijing, Shanghai and Guangzhou. Tier 1 cities are large, densely populated urban metropolises in China with major economic, cultural and political influence. The average income level in these cities is much higher than the national average income. Two general hospital administrators from Beijing were interviewed to provide information on the financial costs of AD. Both experts were selected based on their general experience of managing the price of hospital services and the invoicing process, and dealing specifically with accounting and administrative matters associated with AD in their healthcare institution. For the Delphi consensus meeting, six experts from the previous interviews convened in person to form the discussion panel, which included four physicians and the two hospital administrators.

### Preparatory interviews—qualitative description of AD management

#### Round 1: clinical management and healthcare resource utilisation

Expert physicians were interviewed individually by trained interviewers in September/October 2013. A questionnaire and a discussion guide were provided to each interviewer, who received training on the disease area and how to effectively conduct the interviews. The questionnaire covered four topics:

1. *Diagnosis and referral process.* These questions aimed to identify the main reasons that motivate undiagnosed patients to visit the physician, the severity of disease at the time of diagnosis and the subsequent referral pathways.
2. *Course of illness.* Interviewees were asked to describe the typical course of disease progression observed for AD patients in their practice.
3. *Patient management.* These questions were concerned with the impact of disease severity on the type of healthcare resources and caregiver support typically required by AD patients.
4. *Impact of specific AD symptoms on patient management.* Interviewees were asked to describe how cognitive impairment, dependency and NPS (such as agitation/aggression) influence the management of AD patients.

Lastly, interviewees were asked to report whether they believed their routine practice was different from the common practice amongst their peers.

#### Round 2: costs associated with AD care

Hospital administrators were interviewed individually by trained interviewers in October 2013. The following topics were discussed: diagnostic procedures (such as positron emission tomography [PET] and biological testing), hospitalisation and reimbursement rates. Interviewees were also asked to report whether they believed the healthcare resources available at their hospital were different from the services routinely provided in similar hospitals. This round of interviews helped to clarify the typical hospital funding and invoicing process for healthcare services in China (results not shown). This evidence was used by the study moderator to inform the discussions held during the Delphi consensus meeting.

### Delphi consensus meeting

A Delphi panel meeting was organised in November 2013, where the experts were provided with an overview of the qualitative interview results, followed by the draft structure of a health economic model developed to assess the clinical and economic outcomes of treating moderate and severe AD patients in China. The health economic model was structured to reflect the qualitative results of the preparatory interviews; the panel of experts was then asked to reach agreement on specific data inputs for the model.

Firstly, the experts were asked to estimate the proportion of moderate AD patients seen at diagnosis in each of four possible health states, based on patients’ functional independence and presence of agitation/aggression: 1. independent and non-agitated/aggressive; 2. independent and agitated/aggressive; 3. dependent and non-agitated/aggressive; 4. dependent and agitated/aggressive. The same categories were applied to severe AD patients, resulting in four additional health states. Secondly, experts evaluated the frequency of utilisation of different healthcare resources for AD patients in each health state over a 6-month period. Only healthcare resources associated with the management of AD were considered in this study. Lastly, experts were asked to reach consensus on the unit cost of each healthcare resource used (in Chinese Yuan Renminbi [RMB]).

A moderator familiar with Delphi panel methodology, AD and health economics facilitated the discussions, which involved all six experts present at the meeting. Each question addressed to the panel was discussed until the answers converged towards consensus, which was typically achieved within two to four rounds of debate.

## Results

### Preparatory interviews—qualitative description of AD management

All interviewed physicians stated that the clinical practice described was representative of their healthcare institution.

1. Diagnosis of AD Patients
  The typical clinical investigation process for AD patients was described to start with registration at the hospital, followed by a cognitive assessment, brain imaging procedures and finally a diagnosis. The vast majority of AD patients were considered to be diagnosed in the geriatric or neurology departments of general hospitals. Patients exhibiting NPS are often first seen by psychiatrists, either in specialised psychiatric hospitals, or in the psychiatric department of general hospitals. However, this pathway was considered to be associated with a risk of misdiagnosis in about one third of cases.
  All physicians agreed that mild AD is under-recognised in China, as most patients only seek treatment once moderate to severe symptoms have developed. This delay in diagnosis particularly applies to patients coming from rural areas and those with lower levels of education. In the opinion of the interviewees, the social stigma associated with AD and a general lack of awareness about dementia are the main reasons behind the delayed diagnosis of this disease in China.
  The main reasons that motivate patients, or their caregivers, to seek diagnosis are memory loss, increased dependency and personality changes (agitation/aggression and other NPS). According to the interviewees, disease severity at diagnosis varied widely across hospitals: an estimated one third to one half of patients are diagnosed with mild AD, while the remaining patients tend to be equally distributed between the moderate and severe disease stages. Neurology departments and specialised psychiatric hospitals reported a higher proportion of severe AD at diagnosis because patients are referred to these services in response to more serious NPS, which manifest in the more advanced stages of the disease [29, 6].
2. Disease Progression
  Patients with mild AD were reported to take on average 3–5 years to reach the moderate disease stage, and a further 2–5 years before progressing to severe AD (Table 1).

**Table 1:** Progression of AD and usual symptoms

Diagnosis	3–5 years after diagnosis	6–9 years after diagnosis	≥10 years after diagnosis
30–40 % patients experience mild cognitive impairments and/or personality changes for 1–2 years before visiting the hospital.	30–40 % patients develop NPS (e.g. anxiety, insomnia, distrust of others)	30–40 % patients present more severe NPS (e.g. aggression and depression)	Almost 100 % patients present severe symptoms
		About 50 % patients dependent on caregivers	About 90 % of patients dependent on a caregiver

NPS: neuropsychiatric symptoms
3. Management of AD Patients after Diagnosis
  The interviewees reported that the majority of AD patients are not hospitalised upon diagnosis, due to the lack of healthcare resources at most general hospitals in China. In contrast, patients who first visit the hospital due to NPS are usually referred to psychiatric departments, where they are more likely to remain hospitalised.
  After diagnosis, patients are followed up on a regular basis (outpatient visits every 2 weeks or once a month). Diagnostic procedures are usually repeated when patients come for their second visit, but not in subsequent appointments. Brain imaging procedures for diagnosis and monitoring vary widely across hospitals. Magnetic resonance imaging (MRI) is the most common procedure, while X-ray computed tomography (X-ray CT) or positron emission tomography (PET) may be performed, but are not routinely used.
  According to the interviewees, the main drivers of healthcare resource utilisation by AD patients are the presence of NPS, such as agitation/aggression, and loss of independence. These symptoms can lead to hospitalisation and aggravate the burden on caregivers. It was also reported that 70–80 % of AD patients develop age-related comorbidities, such as bone fractures, diabetes, chronic obstructive pulmonary disease, heart failure, pneumonia and urinary tract infection. Age-related comorbidities are a frequent cause of hospitalisation and mortality among elderly patients in general. However, it was noted that AD complicates the clinical management of age-related comorbidities, increasing the amount of healthcare resources required to manage those conditions.
4. Caregiver Support
  It was reported that most care is provided by unpaid family members, who provide help with basic needs, such as bathing, eating and dressing. Access to nursing homes is limited by the families’ income, as few people can afford formal caregiving, and by the patients’ symptoms, since patients who suffer from complete loss of autonomy or agitation/aggression are less likely to be accepted in a nursing home. However, most nursing homes only provide basic care, with only a limited number of institutions offering memory/cognitive exercises and rehabilitation. Overall, as AD becomes more severe, not only does the burden of caregiving increase, but it also becomes more difficult to find nursing homes or professional caregivers willing to care for such patients, even at high salaries.

### Delphi panel meeting

1. Distribution of AD Patients at Diagnosis
  The panel of experts estimated the distribution of moderate AD patients at the time of diagnosis across four health states, defined by the level of functional independence and agitation/aggression. The majority of AD patients (70 %) were considered to be independent and non-aggressive at the time of diagnosis (Table 2).

**Table 2:** Distribution of moderate AD patients at the time of diagnosis

	Independent	Dependent
Non-aggressive	70 %	10 %
Aggressive	15 %	5 %
2. Healthcare Resource Utilisation
  The consensus reached on the healthcare resources used by moderate and severe AD patients, according to the level of functional independence and agitation/aggression, is presented in Table 3. Overall, dependent AD patients have a much greater requirement for healthcare resources than independent patients. The causes and duration of hospitalisation were a major topic of debate among the experts. Upon thorough discussion, the expert panel recognised that the majority of hospital visits after AD diagnosis are due to age-related comorbidities: AD patients with comorbidities are more likely to be hospitalised, and for longer periods of time, than non-AD patients with the same conditions. This is particularly true for patients in the moderate and severe stages of disease who suffer from agitation/aggression and/or have lost functional independence. Once this fact was acknowledged, experts were able to reach consensus on the probabilities of hospitalisation. For instance, it was agreed that dependent/aggressive AD patients are more likely to be hospitalised (70–90 % probability) than accepted in a nursing home (0–20 % probability), while the opposite is true for dependent/non-aggressive patients (5–35 % probability of hospitalisation vs. 80 % probability of being accepted in a nursing home; Table 3). The average length of hospitalisation was considered to be 2 months.

**Table 3:** Healthcare resource utilisation

	Independent		Dependent	
Healthcare resource	Non-aggressive	Aggressive	Non-Aggressive	Aggressive
Moderate AD patients				
Hospitalisation^a^	0 %	5 %	5 %	90 %
Nursing home^a^	0 %	20 %	80 %	0 %
Caregiver time^b^	1	3	4	10
Consultations^c^	6	6	6	6
Brain imaging^c^	0	0	0	0
Cognitive assessment scales^c^	1	1	1	1
Biological analysis^c^	1	1	1	1
Severe AD patients				
Hospitalisation^a^	1 %	1 %	35 %	70 %
Nursing home^a^	0 %	20 %	80 %	20 %
Caregiver time^b^	1	3	12	15
Consultations^c^	6	6	6	6
Brain imaging^c^	0	0	0.35	0.35
Cognitive assessment scales^c^	0	0	0	0
Biological analysis^c^	1	1	1	1

^a^Probability of using the healthcare resource over a 6 month cycle. ^b^Number of hours of caregiving required per day. ^c^Number of times the healthcare resource is used.
3. Caregiver Time
  The Delphi panel agreed that the amount of caregiving time required increases as moderate AD patients progress to severe stage of disease, due to the gradual cognitive decline and loss of autonomy (Table 3). AD patients with aggressive behaviours require additional supervision, in order to prevent self-harm or aggression towards others (Table 3).
4. Unit Cost of Healthcare Resources
  The highest healthcare cost was attributed to hospitalisation, estimated as costing up to 30,000 RMB/month, which includes all expenses associated with the treatment of age-related comorbidities and AD complications (Table 4). This was followed by the cost of formal caregiving (6000 RMB/month), provided either in a nursing home or by a professional caregiver, and thirdly by the costs associated with the initial diagnostic procedures (Table 4).

**Table 4:** Unit cost of healthcare resources used by AD patients in urban China

Healthcare Resource	Unit cost
Diagnosis hospitalisation	1600 RMB
Hospitalisation^a^	30,000 RMB/month
Consultation (service charge only)	14 RMB
Biological analysis	500 RMB
MRI	1050 RMB
Cognitive assessment scale	100 RMB
Nursing home	6000 RMB/month
Caregiver salary	6000 RMB/month

^a^Cost of hospitalisation for AD patients, including treatment for any complications or comorbidities (the average length of hospitalisation is 2 months). MRI: magnetic resonance imaging; RMB: Chinese Yuan Renminbi

## Discussion

The results of the Delphi study reported here represent the opinions of a panel of expert physicians and hospital administrators, regarding the clinical management and associated costs for moderate and severe AD patients in urban China. When available evidence on a given topic is lacking or contradictory, the Delphi panel approach provides a validated method of combining expert opinion into group consensus [27]. Consensus does not mean that the correct answer has been found, but rather that a level of participant agreement has been reached [30].

Delphi panel studies have been widely used to develop health economic models in a variety of disease areas [31–35]. In developed countries, the availability of medical insurance claims databases, prescription monitoring programmes, disease registries, epidemiological and cost-of-illness studies mean that Delphi panels are now rarely necessary to obtain the data required to build a health economic model. In countries where such information sources are still scarce, the Delphi panel approach constitutes a valuable method of obtaining expert-validated data. In China, the Delphi method has been used in previous analyses of healthcare policy and resource utilisation [36–41], including a study on the cost-effectiveness of different antipsychotic agents in schizophrenia, where a panel of senior psychiatrists estimated patients’ probability of response to treatment and rates of relapse and hospitalisation [42].

Several studies in China and other countries have shown that physicians (especially general practitioners) lack confidence and expertise in diagnosing and managing AD [43–45]. To ensure that the information obtained in this study reflected the level of care provided to patients diagnosed with AD, we decided not to perform a random participant recruitment. Although input from general practitioners would have provided a more complete picture of AD care in China, this was beyond the intended scope of our study. For these reasons, we selected experts with a proven track record in the field of dementia, who are responsible for the clinical management of a large number of moderate and severe AD patients. Therefore, the results of this study reflect the clinical practice performed in the healthcare institutions represented by the invited experts. Although only two hospital administrators participated in the study, the physicians recruited were also very well-informed about Chinese healthcare policies, patient reimbursement schemes and the cost of diagnostic procedures and other healthcare services. Although the experts’ opinions were occasionally different, consensus was easily reached upon debate during the Delphi panel meeting. This suggests that, concerning AD management, there are considerable similarities in terms of healthcare policies and clinical practice across hospitals from tier 1 cities. Indeed, the heterogeneity of clinical practice in China is caused mainly by a healthcare system that applies dissimilar policies to different city levels [26].

All experts interviewed in this study agreed that patients with mild AD remain largely under-recognised in China. This is in line with a recent report suggesting that the majority of individuals with dementia in China are not formally diagnosed, especially in less developed cities and rural areas [9]. The majority of the population believes that the early signs of AD are a normal result of the ageing process, and feelings of shame are common amongst relatives of AD patients [10]. As a consequence, most families only seek diagnosis once patients have lost their autonomy and/or experience symptoms of moderate to severe AD.

The presence of comorbidities, aggressive symptoms and loss of functional independence were considered by the Delphi panel to be the main determinants of healthcare resource utilisation. Since China still lacks adequate healthcare resources and infrastructure for the care of dementia patients [8, 46], the management of AD is a serious issue. For instance, nursing homes providing specialised dementia care are still very rare in China, except in major urban centres [47, 48]. Although severe, dependent and aggressive AD patients require continuous care, they were considered much less likely to be accepted in a nursing home than non-aggressive or independent severe AD patients, a situation that has been previously reported in a multinational Delphi study [41].

Since caring for the elderly is viewed as the family’s responsibility in China, most AD patients receive only informal care from family members [8, 10, 49], which implies that a growing proportion of China’s economically active population will be responsible for the care of dementia patients [50]. As the disease progresses, an increasing amount of time is spent by caregivers looking after the patients, often at the expense of their own health, well-being and financial stability [49, 51, 52]. Recent socioeconomic changes threaten to exacerbate the situation. The migration of young adults to tier 1 cities (such as Beijing, Shanghai and Guangzhou) is predicted to result in large numbers of elderly people being left behind to live on their own. Consequently, an increasing number of patients will depend on formal caregiving services, which are only starting to emerge in China [50, 26]. In spite of these facts, experts in the field of dementia are yet to agree on how to adequately quantify the cost of informal caregiving. Consequently, we were unable to obtain reliable cost estimates for this important component of the societal burden of AD in our discussions with the expert panel. Instead, we have explored the cost of professional vs non-professional care in a separate publication, describing the cost-effectiveness of pharmacological treatment in AD [28].

The duration and cost of hospitalisation was one of the most debated topics in the Delphi panel meeting. Once hospitalisation was agreed to include any AD complications and age-related comorbidities, the experts were able to reach consensus on the average duration and unit cost of this healthcare resource. Furthermore, age-related comorbidities were recognised as the main cause of hospitalisation, a fact that has been previously reported [53, 54]. Patients without AD typically receive treatment for most comorbidities in an out-patient setting. In contrast, AD patients with the same comorbidities are more likely to be hospitalised, and for longer periods of time, due to their cognitive impairment and loss of independence. Consequently, the cost of hospitalisation is much higher for AD patients than for non-AD patients with the same comorbidities.

Although this study provides valuable information about the clinical management of moderate and severe AD in China, there is a need to consider its potential limitations. Given that the experts selected for the Delphi panel were recruited from tier 1 cities, the results of this study apply predominantly to AD patients living in urban areas of China. At present, there are still substantial economic and infrastructural disparities in China between economically developed regions and the rest of the country [26]. Urban populations benefit from higher average income levels, better access to healthcare facilities and tend to spend more on medical services than rural residents [48]. Thus, the rate of AD diagnosis and the healthcare costs we reported are not representative of smaller cities and rural communities, meaning that a substantial proportion of the AD patient population in China was not included in our analysis. However, the heterogeneity of care provided in rural areas would have made it very difficult for the Delphi panel to reach consensus. Since our objective was to obtain data inputs for a health economic model measuring the clinical and economic outcomes of treating AD pharmacologically, restricting this study to urban AD patients was considered an acceptable limitation. We are also aware that limiting our analysis of healthcare costs to the moderate and severe stages of disease provides an incomplete picture of the economic and societal burden of AD. Additional studies are required to define the amount and cost of healthcare resources used by mild AD patients in China. Lastly, it should also be noted that the experts present at the Delphi panel meeting discussed results face-to-face. While this allowed for in-depth discussions and facilitated consensus, it may have inhibited participants from sharing opinions perceived as controversial or different to those of the wider group.

The cost estimates provided in this study were used in a health economic model evaluating the cost-effectiveness of pharmacological treatment for moderate and severe AD patients in China [28]. For this reason, we only considered the cost of healthcare resources associated with AD management and did not include indirect costs, such as travel expenses and loss of productivity by patients and informal caregivers [4], which can exceed the cost of medication and other healthcare resources [25, 52]. Consequently, the costs reported in this study should be viewed as low-end estimates and may not represent the full economic burden of the disease. Nevertheless, the consensus obtained in the Delphi meeting represents the varied clinical and academic experience of the expert panel and provides a valuable depiction of everyday clinical practice in urban Chinese hospitals regarding the care of AD patients.

## Conclusions

The Delphi panel approach provided an efficient method for collecting data and achieving consensus on the clinical management and associated costs for AD patients in urban China. The experts agreed that most AD patients in urban China are diagnosed with moderate to severe disease. Loss of independence, presence of agitation/aggression and age-related comorbidities are the main drivers of healthcare resource utilisation and heavier caregiver burden. The majority of direct medical costs were attributed to hospitalisation and formal caregiving. Due to an ageing population, the cost of dementia-related care already represents a major burden on the Chinese economy. The results of this study provide a valuable source of information for future health economic models of AD in China. Such models will help decision makers to improve healthcare resource allocation and meet the needs of AD patients in a country where dementia is a major public health priority.

## Abbreviations

AD: Alzheimer’s disease
CT: Computed tomography
MRI: Magnetic resonance imaging
NMDA: N-methyl-D-aspartate
NPS: Neuropsychiatric symptoms
PET: Positron emission tomography
RMB: Chinese Yuan Renminbi
USD: United States Dollar
